# Dynamics of ACTH and Cortisol Secretion and Implications for Disease

**DOI:** 10.1210/endrev/bnaa002

**Published:** 2020-02-15

**Authors:** Stafford L Lightman, Matthew T Birnie, Becky L Conway-Campbell

**Affiliations:** Translational Health Science, Bristol Medical School, University of Bristol, Bristol, UK

**Keywords:** cortisol, glucocorticoid, GR, MR, HPA axis, circadian, ultradian, rhythms, dynamics

## Abstract

The past decade has seen several critical advances in our understanding of hypothalamic–pituitary–adrenal (HPA) axis regulation. Homeostatic physiological circuits need to integrate multiple internal and external stimuli and provide a dynamic output appropriate for the response parameters of their target tissues. The HPA axis is an example of such a homeostatic system. Recent studies have shown that circadian rhythmicity of the major output of this system—the adrenal glucocorticoid hormones corticosterone in rodent and predominately cortisol in man—comprises varying amplitude pulses that exist due to a subhypothalamic pulse generator. Oscillating endogenous glucocorticoid signals interact with regulatory systems within individual parts of the axis including the adrenal gland itself, where a regulatory network can further modify the pulsatile release of hormone. The HPA axis output is in the form of a dynamic oscillating glucocorticoid signal that needs to be decoded at the cellular level. If the pulsatile signal is abolished by the administration of a long-acting synthetic glucocorticoid, the resulting disruption in physiological regulation has the potential to negatively impact many glucocorticoid-dependent bodily systems. Even subtle alterations to the dynamics of the system, during chronic stress or certain disease states, can potentially result in changes in functional output of multiple cells and tissues throughout the body, altering metabolic processes, behavior, affective state, and cognitive function in susceptible individuals. The recent development of a novel chronotherapy, which can deliver both circadian and ultradian patterns, provides great promise for patients on glucocorticoid treatment.

## Graphical Abstract

**Graphical Abstract:**
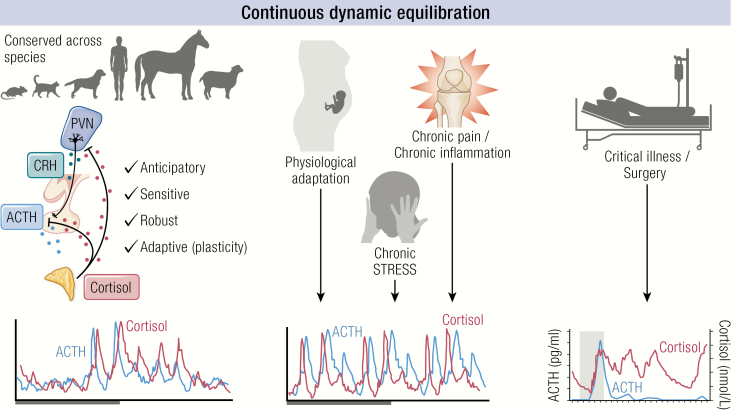

**Essential points**

- Pulsatile glucocorticoid production arises due to a subhypothalamic pulse generator and is the intrinsic property of the feed-forward feedback interplay between the pituitary and adrenal glands
- The pulsatile hormone signal is decoded at the cellular level by the intracellular glucocorticoid receptor (GR), the mineralocorticoid receptor (MR), or both GR and MR in cell types where the 2 are coexpressed
- Long-standing models of GR and MR working in collaboration and in opposition have retained their validity for the most part, with recent evidence for a role of MR in increasing GR transactivation potential during pulsatile glucocorticoid treatment, via a tethering mechanism
- Pulsatile glucocorticoids have now been demonstrated to be required for optimal HPA physiological responses, stress-coping behavior, complex cognitive processing, glutamatergic neurotransmission, synaptic metaplasticity, and emotional processing in man and experimental rodents
- As glucocorticoid ultradian dynamics are altered in myriad disease states, as well as during synthetic glucocorticoid treatment and glucocorticoid replacement therapy, the resulting effects of GR- and MR-expressing cells and tissues can induce detrimental effects on physiological, cognitive, and behavioral function
- Strategies to normalize circadian and ultradian endogenous glucocorticoid rhythms—along with more refined chronotherapies that are able to integrate both circadian and ultradian rhythms into their design—are currently being developed and show great promise

The hypothalamic–pituitary–adrenal (HPA) axis is a neurohormonal system that is critical for life. It is a multisystem axis that utilizes feed-forward and feedback loops to regulate glucocorticoid hormone levels within the physiological range appropriate for system homeostasis. This is an equilibrium control system we have called continuous dynamic equilibration (1). This system has widespread effects in many body systems and not only regulates circadian metabolic, cognitive, cardiovascular, and immunological behavior, but is also vital for protective responsive to both internal and external stressors. In order to fulfil these many disparate roles, the HPA axis needs several hallmark features. These include (1) anticipatory activation to prepare the animal for the active phase of the day (daytime for man and night time for nocturnal animals including most rodent species). It also needs to be (2) *sensitive* to environmental perturbations, and to be able to respond differentially to small and large stimuli. This responsiveness must be (3) *robust* with preservation of dynamic behavior during these perturbations. Finally the system must show (4) plasticity to facilitate adaptation to new circumstances. This concept of dynamic regulation in endocrinology refines the older concept of homeostasis toward a steady-state set point to a more dynamic understanding of how systems oscillate around an equilibrium position and how this allows for a reactive and adaptive system. Furthermore, it provides a conceptual basis for how *allostasis—a* new dynamic equilibrium position in response to novel circumstances—can lead to physiological change and disease. With these thoughts in mind our review will bring together new concepts of continuous dynamic equilibration, and how these provide the basis for understanding the importance of both circadian and ultradian rhythmicity for a responsive and adaptive HPA axis.

## Organization of the Hypothalamic–Pituitary–Adrenal axis

**Figure 1.**
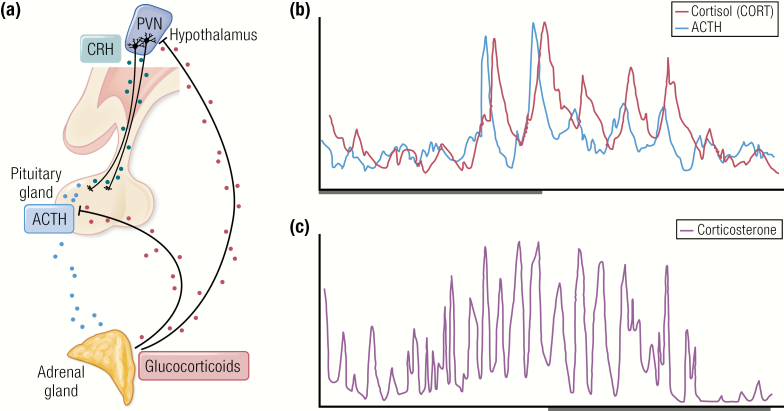
The HPA axis and its hormonal output over the day. (A) A schematic of the HPA axis. CRH (and AVP) are secreted from the PVN. These hormones in turn, stimulate the secretion of ACTH from the anterior pituitary, which in turn, drives the secretion of glucocorticoids from the adrenal cortex. Automated blood sampling has enabled high resolution measurements of the circadian and ultradian profile of (B) ACTH and cortisol (CORT) in human over a 24-hour period and (C) corticosterone in rat.

Within the hypothalamus, the parvocellular neurons of the paraventricular nucleus (PVN) are a group of densely packed neurons that are highly responsive to external physiological stimuli such as altered light/dark cycle, or the presence of real or perceived stress (2,3), as shown in Fig. 1. These cells project to the capillaries of the median eminence, where they secrete corticotropin-releasing hormone (CRH) (and AVP) directly into the portal system and thence pituitary corticotrophs to regulate adrenocorticotropin (ACTH) secretion. Other parvocellular preautonomic neurons project to the brainstem and spinal cord, to regulate appetite and autonomic functions and suppress nociception, promoting analgesic effects (4). Magnocellular neurons of the PVN project directly to the posterior pituitary to release both vasopressin and oxytocin into the systemic circulation (5,6).

CRH and AVP released by parvocellular neurosecretory cells into hypothalamic capillaries that join infundibular blood vessels and reach a second capillary bed in the anterior pituitary to target anterior pituitary corticotroph cells where they stimulate the release of ACTH. This travels in the systemic circulation to reach the *zona fasciculata* of the adrenal cortex to activate the synthesis and subsequent release of glucocorticoid hormones (7).

## Dynamics Within the HPA Axis in Health

An ultradian pattern of cortisol release in humans has been widely reported (8–12). More recently, the development of an automated sampling system for use in humans has enabled blood sampling at a higher frequency than has been previously possible (13). Fig. 1B shows a 24-hour profile of ACTH and cortisol in a healthy volunteer. With 10 minutes of sampling resolution, a short delay is evident between ACTH and cortisol secretion, with each cortisol pulse closely following each ACTH pulse. In healthy male subjects, Russell et al. showed that both ACTH and cortisol pulsatility was rapidly inhibited by intravenous infusion of the synthetic mixed glucocorticoid agonist prednisolone (14). The site of the rapid inhibition of ACTH secretion appears to be the anterior pituitary, as prednisolone also inhibited the ability of exogenous CRH to induce increased ACTH and cortisol secretion. Prednisolone fast feedback could be reduced by pretreatment with the glucocorticoid receptor (GR) antagonist mifepristone but not with the mineralocorticoid receptor (MR) antagonist spironolactone. The rapid dynamics of negative feedback on ACTH secretion were consistent with the plethora of evidence for a ligand-dependent nongenomic GR-mediated negative feedback in the anterior pituitary (15). The pituitary is not the only site where rapid nongenomic negative feedback is found, as Tasker and colleagues have elucidated a mechanism of glucocorticoid suppression by the hypothalamic endocannabinoid system (16–18).

This circadian and ultradian rhythm of adrenal glucocorticoid secretion has not only been recorded in man, but also in every other species tested so far, including rat (19–24) as shown in Fig. 1C, rhesus monkey (25–27), hamster (28,29), horse (30), sheep (31–33), and goat (34–36). A rodent automated blood sampling system designed to perform frequent sampling on freely behaving rats in their home cage environment (1,37) has revealed the ultradian rhythm of rats in great detail, showing distinct pulses at approximately hourly intervals. The ultradian corticosterone rhythm of rats has been found to exhibit significant sex differences (38–41), and is subject to further change during lactation and aging (42–44), as a result of early life stress (45) and inflammatory disease (46,47). The HPA axis has also been found to exhibit remarkable plasticity associated with physiological changes throughout life in healthy humans. An example of HPA axis adaptation to rapidly changing physiology occurs during pregnancy. The maternal HPA axis undergoes dramatic activation during pregnancy resulting in increased circulating cortisol, and a study at the Edinburgh Royal Infirmary was able to quantitatively assess this phenomenon using multiple peripheral blood and 24-hour interstitial fluid samples on 5 healthy pregnant women at 16 to 24 weeks’ gestation (P1) and again at 30 to 36 weeks’ gestation (P2) compared with a control group of healthy nonpregnant (NP) women. While an observed increase in cortisol pulse amplitude was not significantly different (NP 44 nmol/L, P1 99 nmol/L, P2 131 nmol/L; *P* = .09), significant differences were found in the increased fasting serum cortisol (NP 302nmol/L, P1 528 nmol/L, P2 779 nmol/L; *P* = .018) as well as in the increased frequency of the cortisol pulses (NP 1.1 pulses/hour, P1 1.5 pulses/hour, P2 1.6 pulses/hour *P* < .0001) (48). As human automated sampling methodologies become more refined (49,50), there will be far more detailed information about which features of the HPA axis are conserved between humans and experimental rodents, and where species specific differences exist.

There are very clear similarities in the regulation of HPA dynamics found in experimental rodent studies and clinical studies in man. Consistent with the dynamics of the pituitary adrenal system observed in man (13), each pulse of ACTH is followed by a pulse of corticosterone in the rat (35). Similar to the human study findings, exogenous glucocorticoids exert a rapid inhibition of both basal and CRH-induced ACTH and corticosterone secretion (51–55) indicating the anterior pituitary as the primary site of rapid negative feedback.

### The pulse generator

**Figure 2.**
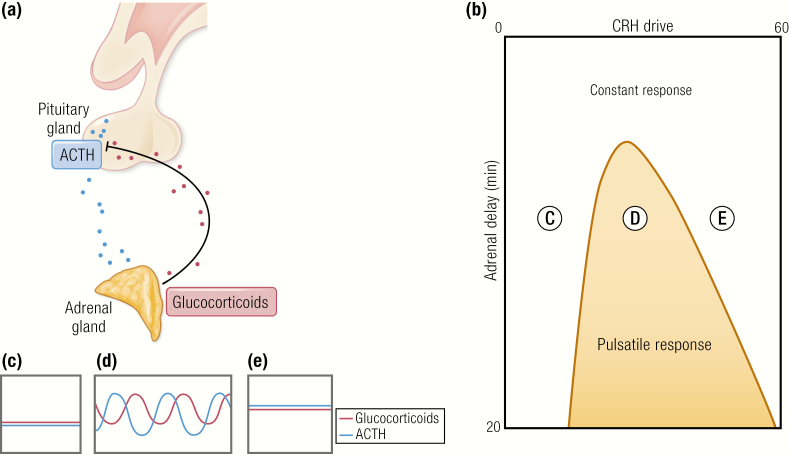
Response of the pituitary–adrenal system to constant CRH drive. (A) Feed-forward feedback interplay between the pituitary and adrenal accounts for ultradian oscillations in glucocorticoid secretion. (B) Different combinations of constant CRH drive and delay can lead to 2 qualitatively different responses. On one side of the transition curve, when the CRH drive is low, the pituitary–adrenal system responds with constant levels in ACTH and glucocorticoid (C). On the other side of the transition curve, the pituitary–adrenal system responds with pulsatile fluctuations in the levels of ACTH and glucocorticoid, despite the fact that the CRH drive is constant (D). On the far right-hand side of the transition curve, when the CRH drive is highest, the pituitary–adrenal system again responds with constant levels in ACTH and glucocorticoid (E). Model predictions for ACTH (blue) and glucocorticoid (pink) are shown in C, D, and E. Walker et al. (57).Walker JJ, Terry JR, Lightman SL. Origin of ultradian pulsatility in the hypothalamic-pituitary-adrenal axis. Proc Biol Sci 2010;277:1627–1633. CC-BY OA. © 2010 Springer Nature.

The general notion of a hypothalamic pulse generator prevailed until quite recently (56), despite strong evidence to the contrary from elegant studies in sheep which demonstrated ultradian pulses of ACTH and cortisol persisted after hypothalamic pituitary disconnection (33). Walker et al. (57) used their rodent data to come up with a mathematical model that concluded endogenous glucocorticoid pulses must arise due to the intrinsic relationship between the feed-forward signal from the anterior pituitary to the adrenal and the feedback signal from the adrenal to the anterior pituitary. Fig. 2 shows the model predicting that even a constant input of CRH will result in oscillations of ACTH and glucocorticoids, due to the inherent delay which exists in both the forward part and the reverse part of the loop. This was tested with a constant CRH infusion in the rat (58). As predicted by the model, ACTH and corticosterone pulsatility could be experimentally reinstated without a pulsatile CRH signal. Instead, pulsatile ACTH and subsequent pulsatile corticosterone was found to be entirely dependent upon the level of CRH rather than the pattern, as predicted by the mathematical model. Very low level CRH infusions were unable to induce any detectable activity, while very high CRH levels produced disrupted pulsatile pituitary adrenal activity. Constant CRH infusion, at a dose that was more closely matched to physiological circadian peak levels, was able to induce ACTH and corticosterone oscillations with the time delay predicted by the mathematical model.

### Dynamic regulation at the systems level

Although the pituitary–adrenal interaction is the mechanism underlying ultradian rhythmicity, hypothalamic modulation of the HPA axis is the dominant factor regulating pulse amplitude over the course of each day. Circadian rhythms in activity, body temperature, and hormonal systems are tightly controlled by the central circadian pacemaker, the suprachiasmatic nucleus (SCN) in the hypothalamus, which receives light cues via the retinal projections to entrain to a 24-hour rhythm (59,60). Consistent with this, circadian modulation of the amplitude of corticosterone pulses was profoundly affected by SCN lesion and constant light exposure in rats (61). Notably, the circadian nadir appeared to become “disinhibited” in a manner that was consistent with the described role for the SCN in HPA inhibition during the light phase in the nocturnal rat. In rat, GABAergic interneurons exert inhibitory tone on the PVN to decrease CRH release during the circadian nadir. In contrast, in diurnal species such as man, increased CRH release is hypothesized to be the result of excitatory input from glutamatergic interneurons (62,63). The SCN has also been shown to directly modulate adrenal sensitivity via autonomic nervous system innervation by the splanchnic nerve. Elegant experiments by Jasper and Engeland in the 1990s demonstrated that splanchnic denervation in the rat resulted in a loss in the circadian glucocorticoid nadir (22) with high amplitude pulses throughout the day, similar to that observed in SCN lesioned rats. Splanchnic denervation also increased the adrenal response to ACTH (24). These data taken together strongly indicate that the neural pathway, mapped in rat, from the SCN via the autonomic PVN and splanchnic nerve to the adrenal gland is inhibitory. There also appears to be a more direct effect of light on corticosterone secretion which is wavelength dependent (64).

### Intra-adrenal dynamics

One of the key properties of the pituitary adrenal interaction is the inherent delay in ACTH-induced glucocorticoid release from the zona fasciculata of the adrenal cortex. This is because glucocorticoid hormones, lipophilic molecules, cannot be stored in vesicles but require de novo synthesis by steroidogenesis. For each pulsatile secretory event, steroidogenesis is initiated by ACTH binding to the cell surface G-protein coupled melanocortin type-2 receptor (MC2R) (65), which activates a signaling cascade of 3′,5′-cyclic AMP, protein kinase A, hormone-sensitive lipase, and steroidogenic acute regulatory protein (StAR), resulting in transfer of cholesterol to the inner mitochondrial membrane where it is rapidly converted to glucocorticoids via a series of enzymatic conversions (66). The first and rate-limiting enzyme in this process is cytochrome P450 side chain cleavage enzyme (67,68), which catalyzes the conversion of cholesterol to pregnenolone for subsequent rapid conversion steps through progesterone to 11-deoxycortisol to cortisol, or alternatively through progesterone to 11-deoxycorticosterone to corticosterone. In humans, cortisol is the predominant glucocorticoid produced. In rats, corticosterone is the predominant glucocorticoid produced instead of cortisol, due to the lack of 17 alpha-hydroxylase (66).

**Figure 3.**
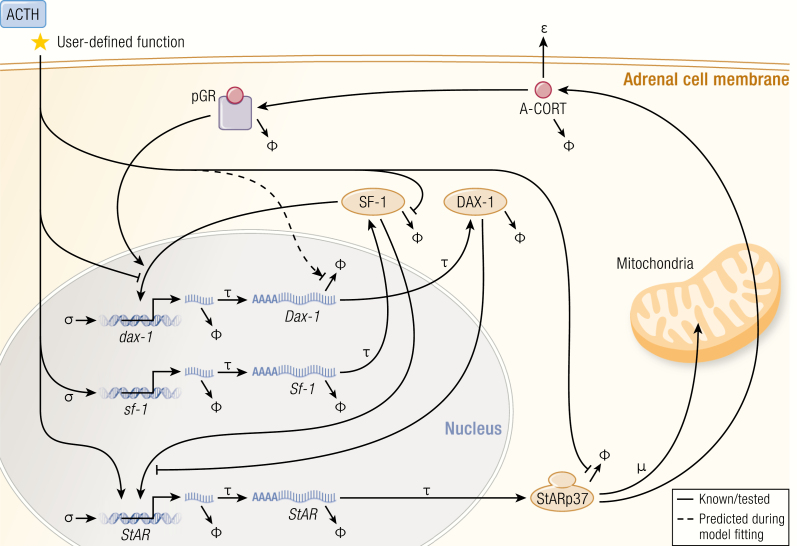
The steroidogenic regulatory network. The synthesis of glucocorticoids in adrenocortical cells is governed at multiple levels by both genomic and nongenomic components indicated in this schematic. In order to constrain the complexity of the model only nodes shown to be involved in glucocorticoid-mediated feedback loops or in crosstalk with StAR are included. The model therefore consists of a set of delay differential equations (DDEs) that describes the ACTH stimulated dynamics of intra-adrenal glucocorticoid (A-CORT) levels and phosphorylation of GR (pGR, a marker of GR activation), and the expression of DAX-1, SF-1, and StAR. Symbology: σ accounts for the basal non-ACTH-dependent gene promoter activation rate. It is also more commonly known as “leaky transcription”. τ represents time delays, in this case, transcription and translation of each gene. ϕ represents degradation sinks for each molecular species, in this case heteronuclear RNAs (hnRNAs), mRNAs, and proteins. The model predicted that ACTH should modulate the half-life (stability or degradation rate) of Dax-1 mRNA, depicted here by the dashed line, and required in order to explain the data. μ represents a combination of 2 processes: the proteasome-mediated degradation of StARp37 as it progresses through the outer to inner mitochondrial membrane, and the import of StARp32 and StARp30 into mitochondria. ɛ represents the export rate of intra-adrenal glucocorticoid (A-CORT) out of adrenocortical cells. Spiga F, Zavala E, Walker JJ, Zhao Z, Terry JR, Lightman SL. Dynamic responses of the adrenal steroidogenic regulatory network. Proc Natl Acad Sci U S A 2017; 114:E6466-E6474. CC-BY OA © 2017 Springer Nature.

Spiga et al. have elucidated a complex regulatory network, which acts together with cAMP response-element binding protein (CREB), notably including the positive regulators SF-1 and Nur77, and the negative regulator Dosage-sensitive sex reversal, adrenal hypoplasia congenita, critical region on the X chromosome, gene-1 (DAX-1) as shown in Fig. 3 (69). Elegant time course studies have shown, both in vivo (69) and in zona fasciculata (ZF) model cell lines ATC1 and ATC7 (70), that the phosphorylation of steroidogenic proteins follow an ultradian rhythm. Pulses of ACTH can induce pulses of corticosterone secretion in HPA axis-suppressed rats, whereas the same dose delivered in a constant infusion fails to induce pulsatile corticosterone secretion (71). The mechanism was further explored in the ZF cell lines (70) where it was found that constant ACTH treatment induced larger increases in pCREB and steroidogenic gene transcription at the start of treatment but the cells became unresponsive to the stimuli over time. Continuing responsiveness over several hours was achieved with pulsatile ACTH application, further supporting the conclusion that pulsatile ACTH is required for optimal regulation of steroidogenesis in adrenal ZF cells. In addition to this rapid steroidogenic pathway for synthesizing glucocorticoids in pulses, ACTH also induces transcription of many genes including *MC2R*, *StAR*, and *CYP11A1* (the gene encoding cytochrome P450 side chain cleavage enzyme) to presumably replenish the cellular store of steroidogenic pathway components.

There is also an inherent short delay in the negative feedback part of the loop. Once synthesized, glucocorticoids rapidly travel through the circulation to inhibit the HPA axis at the level of the hypothalamus and anterior pituitary, inhibiting synthesis and release of CRH (72–75) and ACTH (76–78), respectively. Recent data have further implicated a novel intra-adrenal negative feedback loop involving GR, which may rapidly inhibit ACTH-induced steroidogenesis (79). Consistent with this hypothesis, Spiga et al. (69) have shown a rapid and transient activation of adrenal GR with each pulse of corticosterone synthesis. Interestingly, GR and SF-1 have been demonstrated to work together to increase expression of the steroidogenic inhibitor DAX-1, while ACTH disrupts this GR/SF-1 interaction (80). Therefore, the opposing actions of ACTH and intra-adrenal corticosterone on DAX-1 are hypothesized to also contribute to the rapid oscillations in intra-adrenal steroidogenesis.

## Decoding the Glucocorticoid Oscillating Signal at the Cellular Level

Endogenous mammalian glucocorticoid actions are mediated via 2 corticosteroid receptors. The high-affinity MR originally termed the type 1 corticosteroid receptor is encoded by the *NR3C2* gene (81). The low-affinity GR originally termed the type 2 corticosteroid receptor is encoded by the *NR3C1* gene (82).

**Figure 4.**
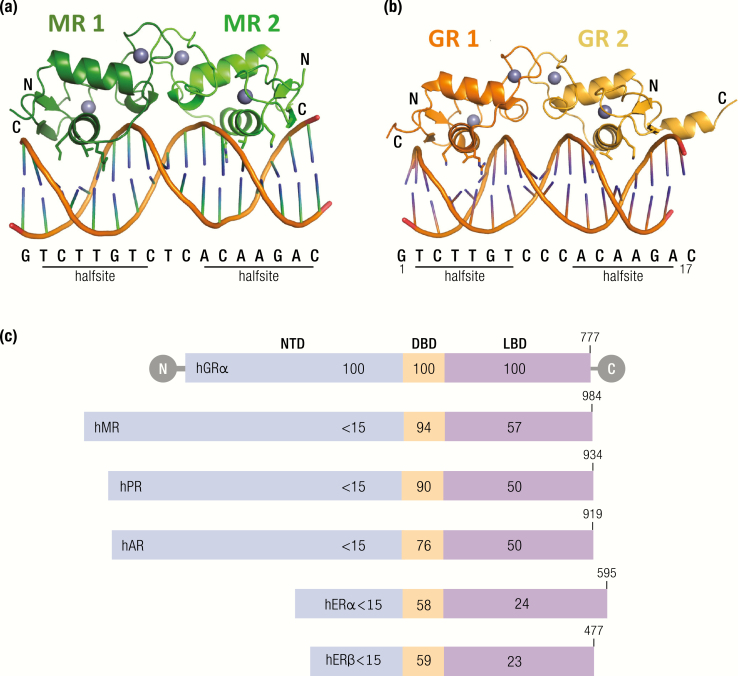
Comparison of MR or GR homodimers bound to a GRE. The sequence of the element, along with the 2 bound half sites, is shown below the structure. (A) The structure of 2 MR DBDs (each monomer depicted in a different shade of green) bound to a 17 base pair GRE shows the asymmetric unit of the crystal structure (84). (B) The structure of the GR DBD (each monomer depicted in a different shade of orange) bound to a similar GRE, with the exception that it is derived from the structure of the GR DBD bound to the FKBP5 GRE (85). (C) The steroid receptors have a highly conserved protein domain structure. The % sequence identity is indicated for each domain, relative to hGR. The size of each protein is indicated on the schematic; hGRalpha 777 amino acids, MR 984 amino acids, PR 934 amino acids, AR 919 amino acids, ERalpha 595 amino acids, ERbeta 477 amino acids. Abbreviations: NTD, N-terminal domain; DBD, DNA binding domain; LBD, ligand binding domain. GR and MR have a highly conserved DBD at 94% similarity. The LBD is also more similar between GR and MR than GR and other members of the steroid receptor family although at 57% identity there are some important structural and functional differences in ligand binding affinity and specificity. The NTD is the least similar between members of the steroid receptor family. AdaHudson WH, Youn C, Ortlund EA. Crystal structure of the mineralocorticoid receptor DNA binding domain in complex with DNA. PLoS One 2014;9:e107000. [Adapted under Open Access License.]

GR and MR are classed with the steroid hormone receptors, which are a conserved subset of the nuclear receptor superfamily (83). Nuclear receptors are diverse in their functional output, controlling homeostasis, metabolism, and development. These receptors are ligand activated transcription factors, and following activation by hormone binding, the receptors bind to regulatory deoxyribonucleic acid (DNA) regions of target genes to initiate transcriptional activation or repression. Nuclear receptors share sequence and structure similarity and comprise 3 major functional domains as shown in Fig. 4: The N-terminal domain (NTD), which contains the activation function domain 1 (AF1), and acts in a ligand-independent manner. A central DNA binding domain (DBD) and a C-terminal ligand-binding domain (LBD), containing the ligand-dependent activation function 2 (AF2) site, which is tightly regulated by hormone binding. The LBD is connected to the NTD by a hinge region, enabling the receptors to act as ligand-activated transcription factors, binding to specific DNA sequences and recruiting other factors via the AF sites to evoke a high level of transcriptional regulation.

The LBD consists of 12 helices that fold into a globular structure, each consisting of 3 sets of helices forming the sides and top of the globule, creating a central hydrophobic pocket where the ligand can bind. This structure is held in an open configuration by the association of chaperone proteins. The DBD contains 2 zinc finger motifs which are responsible for recognition and binding of both MR (84) and GR (85) to target regions of DNA known as glucocorticoid response elements (GREs). The first zinc finger comprises a P-box with a glycine, serine, and valine that interact with specificity to the GRE. The second zinc finger is required for receptor dimerization. In vitro studies have previously found GR binds as an inverted dimer to 2 6 bp palindromic sequences, separated by a 3 bp spacer. These 2 zinc fingers can therefore work together to promote GR binding with the first zinc finger binding to the first half of the palindromic sequence, and the second zinc finger enabling the binding of another GR to the other half of the palindromic sequence. Binding of a homodimer to the GRE of specific genes can promote transactivation. The NTD is the least well conserved domain but contains the ligand-independent AF1 site. AF1 in this region has been shown to communicate with coactivators, chromatin modulators, and basal transcription factors including RNA polymerase II and TATA binding protein to initiate transcription. In the GR, the AF1 site remains relatively unfolded in the basal state, while it forms a significantly complex helical structure in response to binding to cofactors, including TBP and p160 coactivators (86).

The type 1 and type 2 corticosteroid receptors (MR and GR) were both cloned by 1987 (81,82) and were found to be very similar in structure and function. Notably, there is 94% DBD identity, and consequently the 2 receptors recognize and bind the same DNA regulatory sequences, termed glucocorticoid regulatory elements (GREs). There is 57% similarity in the LBD, with consequent functional implications for differences in MR and GR ligand affinity and specificity. There has therefore been great speculation about MR versus GR specificity, particularly in tissues such as the hippocampus, where they are both abundantly expressed and often colocalized.

### Ligand binding and activation dynamics

**Figure 5.**
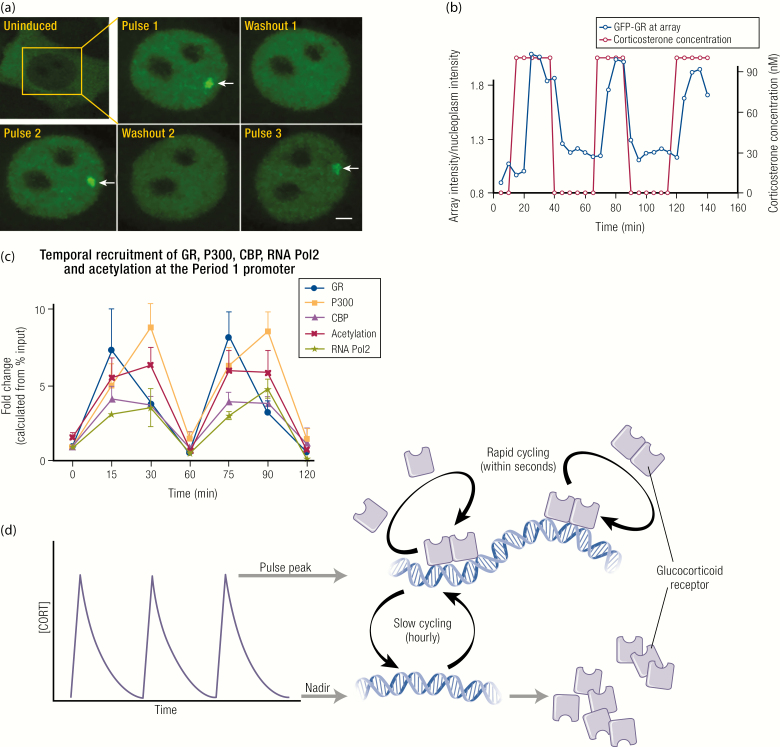
Key concepts underpinning the GR ultradian cycling model. (A) Pulsatile GFP-GR recruitment and loss from the MMTV array is visualized in real time during pulsatile glucocorticoid (CORT) addition to the MMTV array containing cell line. (B) Fluorescence intensity at the array relative to fluorescence intensity of the surrounding nucleoplasm is quantified and plotted in blue over timing of corticosterone (CORT) pulse addition to the cell culture media plotted in red. Stavreva DA, Wiench M, John S, Conway-Campbell BL, McKenna MA, Pooley JR, Johnson TA, Voss TC, Lightman SL, Hager GL. Ultradian hormone stimulation induces glucocorticoid receptor-mediated pulses of gene transcription. CC-BY OA Nat Cell Biol 2009;11:1093–1102. © 2009, Springer Nature. (C) The temporal dynamics of the system has been interrogated in the pituitary cell line AtT20, where pulsatile recruitment of GR, P300, CBP, and RNA-Pol2 to the Per1 proximal GRE is relative to the transient pulses of acetylation at the same site. Conway-Campbell BL, George CL, Pooley JR, Knight DM, Norman MR, Hager GL, Lightman SL. The HSP90 molecular chaperone cycle regulates cyclical transcriptional dynamics of the glucocorticoid receptor and its coregulatory molecules CBP/p300 during ultradian ligand treatment. Mol Endocrinol 2011;25:944–954. Reproduced with permission. Mol Endocrinol 2011; 25:944–954 © 2011, Endocrine Society. (D) A schematic representation of GR activity at the chromatin template during pulsatile peak and nadir. At the pulse peak, GR undergoes rapid cycling to efficiently sample GRE sites across the genome. At this time, GR can be detected as “enriched” by ChIP assay at cell-specific target regulatory sites. At the pulse nadir, GR in no longer “enriched” at these same sites when assessed in ChIP assays. Subsequent cycles of GR activity ‘ON’ and “OFF” the chromatin template closely follow the peaks and troughs of each glucocorticoid pulse.

The endogenous glucocorticoids corticosterone and cortisol bind to MR with a 10-fold greater affinity than to GR (87–89). During periods of low circulating glucocorticoid levels (ie, during the inactive phase), which is the daytime for the nocturnal rat, the MR is already substantially occupied but the GR is not. GR becomes activated at higher glucocorticoid concentrations (ie, during the active phase), or during a stress response (90,91). GR and MR also exhibit strikingly different dynamics in response to ultradian glucocorticoid pulses. During the high amplitude pulses of the active phase, the peak of each glucocorticoid pulse transiently induces GR activation, resulting in hourly cycles of association/dissociation from DNA (92–95). Pulses of glucocorticoid have therefore been defined as deterministic for ultradian transcriptional activity, directing ordered recruitment of GR cofactors and the transcriptional machinery to glucocorticoid target genes (96), as shown in Fig. 5.

More extraordinary than the fast rate of GR recruitment of cofactors with the rising glucocorticoid concentration is the rapid ejection of each component of the complex from the chromatin template during the falling phase of each pulse. Dissociation of GR from GREs in the DNA can be most simply explained by rapidly declining glucocorticoid levels during the falling phase of individual pulses. Due to the relatively low affinity of GR for endogenous glucocorticoids, pulse nadir levels are not able to maintain GR activation and DNA binding throughout the interpulse interval. The underlying mechanism for this phenomenon has been addressed in studies into the inherent nature of transcription factor–DNA interactions (97). In contrast to the now outdated cooperative binding model, where a stable complex is formed between transcription factors and DNA, the current evidence based on 2 decades of research supports a much more dynamic exchange of transcription factors at the chromatin template (100–102), the phenomenon termed “rapid cycling.” The development of a murine mammary carcinoma cell line with an expanded mouse mammary tumor virus (MMTV) long terminal repeat (800–1200 GRE sites in 1 locus, termed the MMTV array) enabled the first direct visualization of green fluorescent protein tagged GR (GFP-GR) recruitment to a regulatory DNA site large enough to be observed as a single bright focal point within a cell’s nucleus (101). Fluorescence recovery after photobleaching analysis could then be carried out to determine how rapidly activated GFP-GR molecules exchange at the array (102). Since the array rapidly regained its fluorescence after photobleaching, this indicates a rapid exchange between the existing photobleached GFP-GRs, and GFP-GRs from outside the defined photobleached area. The speed of recovery was consistent with GRs rapidly exchanging at the chromatin template in a matter of seconds (t_1/2_ = 5 seconds), indicating that each GR binding event at the chromatin template occurred for 10 seconds to 20 seconds before exchanging. While GR’s recruitment was deemed to be highly stochastic, its ejection from the chromatin template was found to be dependent upon ATP and remodeling complexes (103,104). Therefore, GRs are believed to be ejected from the DNA template with each cycle of nucleosome repositioning as the chromatin is remodeled, before being stochastically and indiscriminately recruited to any one of the vast number of GRE sites throughout the genome. With each cycle of exchange, there is a probability of ligand loss, leaving GR inactive and unable to reassociate with a GRE during the pulse nadir.

**Figure 6.**
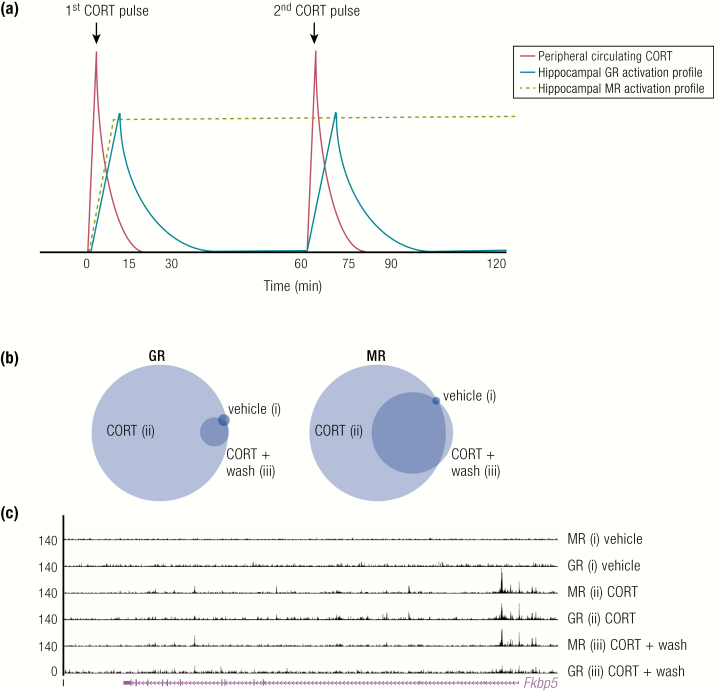
Dynamics of glucocorticoid receptor and mineralocorticoid receptor during ultradian glucocorticoid exposure. (A) Representative plot (adapted from (92)) showing temporal dynamics of hippocampal GR and MR activation times in relation to 2 intravenous corticosterone (CORT) pulses administered to adrenalectomized rats. (B) Area-proportional Venn diagrams show the proportions of GR and MR MACS2 binding sites that directly overlap by at least 1 bp between treatments (i) vehicle, (ii) CORT pulse, and (iii) washout period after CORT pulse. (C) University of California Santa Cruz Genome Browser image at the *Fkbp5* gene shows comparison of mapped MR and GR ChIP-nexus data for each treatment group. Rivers CA, Rogers MF, Stubbs FE, Conway-Campbell BL, Lightman SL, Pooley JR. Glucocorticoid receptor-tethered mineralocorticoid receptors increase glucocorticoid-induced transcriptional responses. Endocrinology 2019;160:1044–1056 (B and C; 108). Reproduced with permission Endocrinology 2019; 160:1044–1056. © Endocrine Society.

In contrast, MR has been demonstrated to remain maximally activated throughout the ultradian interpulse interval (92) as shown in Fig. 6. The prolonged MR activation time is most likely due to a combination of its higher affinity and longer binding duration for endogenous glucocorticoids. While MR has not been reported to undergo rapid exchange at GRE sites, it is likely that it also exhibits this activity as rapid exchange has been shown for all members of the nuclear receptor family tested so far. Presumably, rapid exchange of MR at the DNA template still continues even at much lower endogenous glucocorticoid concentrations. Importantly, the higher sensitivity of MR to glucocorticoids should not be misinterpreted as “constitutive” activity, as DNA binding was undetectable at extremely low glucocorticoid levels, in adrenalectomized rats for example. Interestingly, the MR was previously believed to remain at near maximal ligand occupancy during the circadian peak and nadir (87,88). However, more recent evidence suggests that MR exhibits circadian differences in binding to specific gene target regulatory elements, for example on the *Fkpb5, Per1 and Sgk1* gene. In the presence of an acute stressor, the binding of MR at a regulatory element within *Fkbp5* is also further increased (91,105). A recent pulsatile glucocorticoid study by Rivers et al. (106), in the N2A neuronal cell line, confirmed that MR binding persisted during the interpulse interval at the majority of sites, whilst GR binding was lost at all sites. Additionally, the novel role for MR in augmenting GR’s transcriptional activation potential at the pulse peak was identified in this study. This action of MR did not require its DNA binding, as it was able to be tethered to GRE sites by GR (106), potentially as a heterodimer (91,105,107,108) or other oligomeric form such as a tetramer as proposed by for GR (109).

### Expression profiles of the corticosteroid receptors

The presence of corticosteroid binding sites in the brain was first discovered by Bruce McEwen and colleagues in the late 1960s (110), and a decade of research ensued to characterize these binding sites as 2 separate “populations” that are now known as GR and MR (111,112). It is now well accepted that GR is widely expressed throughout most cells and tissue types in the body. In the brain, GR has been found to be highly abundant in the hippocampus (HC) and medial prefrontal cortex (mPFC), as well as being expressed within the amygdala, areas that are associated with memory and learning processes (87,89,113). The pyramidal cells of the cornu ammonis (CA) 1 and CA2 regions of the HC have been shown to express GR at high levels, as well as the granular cells of the dentate gyrus (114). High levels of GR have also been shown in the cerebellar cortex, olfactory cortex, thalamus, hypothalamus, dorsal nucleus raphe, and locus coeruleus (87,89,115).

MR is generally reported to exhibit a more restricted expression profile throughout the body, with notably high levels of expression in the kidney and adipose tissue (116). In the brain, MR expression has been reported in the prefrontal cortex, the medial and central amygdala, lateral septum, thalamic nuclei, and hypothalamic nuclei (119–121). However, the highest expression of MR has been reported in the CA1, CA2, CA3, and dentate gyrus of the hippocampus (89). However, a recent study in the *Parus major* has shown that MR is prevalent across many areas of the brain including the locus coeruleus and the oculomotor nerve (120), therefore it may still remain to be seen how widespread MR expression is in humans and experimental rodents.

### GR and MR action in the brain—HPA axis regulation

When de Kloet and Reul (87) originally found that MRs were substantially occupied with ligand in basal conditions during the circadian nadir, they hypothesized that MR may have a role in maintaining the HPA axis set point. In 1989, Mary Dallman showed this to be case, reinstating normal hypothalamic and pituitary set points in adrenalectomized rats by replacing corticosterone at a dose that activated MR but not GR (121). The MR antagonist RU28318 administered directly into the brain of rats, via Intracerebroventricular (ICV) or intrahippocampal infusion, increased basal HPA axis activity and potentiated the initial rise in ACTH and corticosterone secretion in response to stress (122,123). In humans, systemic treatment with the MR antagonist spironolactone also increased basal and stress induced cortisol secretion (124). In contrast, GR antagonism with RU38486 had no effect on basal HPA axis activity, which may be expected as GR is not activated by the low glucocorticoid levels secreted during the circadian nadir in basal conditions. The use of RU38486 was however able to elucidate GR’s major role in the response to stress, as antagonist treatment attenuated the initial HPA stress response and therefore resulted in prolonged cortisol secretion due to inhibition of GR negative feedback (122,123).

### Role of pulsatile activity in the stress response

In the late 1990s, Windle et al. (20) noted that his experimental rats’ HPA response to a mild psychological stressor (10 minutes of 99 db of white noise) was variable, with the corticosterone response diverging into 2 distinct groups as “responders” and “non-responders’. Subsequent post hoc analysis of each rat’s corticosterone profile relative to the onset of the noise stress revealed an extremely interesting finding. When the stress coincided with the rising phase of a pulse, adrenal corticosterone secretion was potentiated. When the stress coincided with the falling phase of a pulse, adrenal corticosterone secretion was inhibited. Sarabdjitsingh et al. (125) went on to further interrogate this observation, by introducing exogenous pulses of corticosterone back into adrenalectomized rats and timing the onset of a noise stress to either the rising or falling phase of a pulse. Consistent with Windle’s original findings, the HPA axis response (measured by ACTH production in this case) was potentiated in the rising phase, and significantly attenuated in the falling phase of the infused corticosterone pulse. Not only was the HPA response sensitive to the phase of the pulse, but so too was neuronal activation in the pituitary, paraventricular nucleus, amygdala, and hippocampus, as well as the behavioral coping response to the stressful encounter.

Although it might seem strange that responsiveness of the HPA depends to a degree on an hourly phase, it is easier to think of this in terms of maintaining system responsiveness. Thus in classic pharmacology constant exposure to a ligand often results in downregulation of response, similarly if we compare response parameters between constant infusion of corticosterone of the same dose given in physiological pulses, we find that during the “constant corticosterone infusion,” all 3 stress response parameters measured were significantly impaired in comparison to both rising and falling phase pulsatile. Therefore ultradian glucocorticoid oscillations appear to be critical for the maintenance of normal physiological, neuronal, and behavioral reactivity to stress.

### Learning and memory

The role of glucocorticoids in hippocampal memory function has historically been one of the most well-described effects of glucocorticoids on the brain. We now know that stress-induced glucocorticoids enhance memory consolidation and impair memory retrieval, as well as induce a shift from hippocampal controlled cognitive processing to dorsal striatum controlled cognitive processing. Pharmacological studies in rats originally tested in the spatial memory task of the Morris water maze showed that blockade of GR after learning prevented memory consolidation (126,127). The GR-mediated effects were dependent upon gene transcription and could be observed with systemic, ICV and intrahippocampal site-specific administration of the GR antagonists. Memory consolidation was also abolished in mice with a point mutation in the GR dimerization domain (128).

In the same Morris water maze tests, blockade of MR affected memory retrieval and the selection of behavioral search strategy (126,127). Molecular manipulation of MR by knockdown or overexpression in mice also altered exploratory and searching behavioral strategy in a stimulus response task (131). Interestingly, similar behavioral observations have been made in humans (132–135), where MR plays a role in switching between simple effective strategies and complex flexible strategies when required in different tasks, and particularly in stressful situations. These studies utilized functional magnetic resonance imaging, showing the switch between hippocampal activity and dorsal striatum activity, while tasks of different complexities were undertaken.

**Figure 7.**
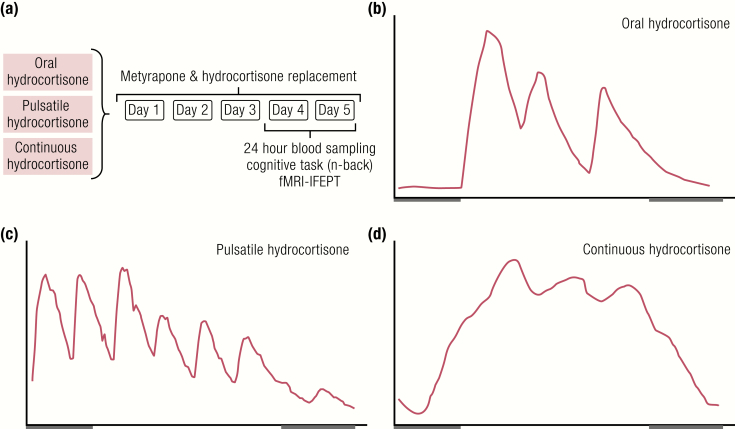
Schematic representation of experimental design and patterns of hydrocortisone replacement. (A) Protocol design and ABS profiles showing the circulating cortisol pattern with either (B) oral hydrocortisone dosing, or subcutaneous pump infusion of (C) pulsatile hydrocortisone or (D) constant hydrocortisone. From (134) and (135).

Taken together, these data from pharmacological, behavioral, and functional imaging studies in rodents and humans highlight an important role for the dual GR system in cognitive processing. However, little was known about the role of the glucocorticoid pattern in memory and learning until a recent study by Kalafatakis and colleagues (134,135). In this study, a double-blinded, placebo-controlled, crossover design in healthy volunteers was used to assess how different patterns of hydrocortisone affect emotional and cognitive processes in these otherwise healthy individuals. Fig. 7 shows a schematic of the experimental protocol. Each volunteer took part in 3 5-day, randomized order, block and replace studies. Endogenous cortisol was blocked with metyrapone and hydrocortisone was replaced using either of 3 options: (1) standard oral dosing, (2) subcutaneous pump delivery with both circadian and ultradian rhythm components, and (3) subcutaneous pump delivery with a circadian rhythm, but no ultradian rhythm.

On the 5th day of each treatment arm, cognitive performance was measured in an n-back working memory task. Interestingly, no significant differences were found between the 3 treatment arms when the cognitive demands of the working memory task were relatively low in the 2-back task. However, when the complexity of the task increased to 3-back, significant differences were revealed in the subject’s ability to retain their working memory capacity. Only during pulsatile replacement could subjects perform well under the increased cognitive demand, indicating a role for optimal pulsatile glucocorticoid replacement in retention of working memory capacity under increased cognitive demands.

### Ultradian glucocorticoid pulses balance glutamatergic transmission and synaptic plasticity

The emerging evidence for rapid nongenomic MR actions (136) combined with slower genomic GR actions (137) provides a physiologically realistic mechanism to explain the dynamic glucocorticoid control of glutamatergic neurotransmission. The effects on neuronal excitability are not only dose dependent, indicating distinct GR- and MR-dependent actions, but also operate in different time domains (137). A series of elegant studies by Marian Joëls’ group (138) showed that a single corticosterone pulse altered baseline transmission during the interpulse interval, by increasing synaptic enrichment of glutamate receptors, altering responsiveness to spontaneously released glutamate, and preventing subsequent LTP induction. A second pulse normalized glutamate transmission and synaptic plasticity. This phenomenon has been termed stress metaplasticity, a mechanism whereby response to glucocorticoid pulses can switch from excitatory to inhibitory depending on the recent “stress” history. Consistent with how vividly emotionally arousing experiences are encoded into memories, neurons in the basolateral amygdala are highly sensitive to stress plasticity (139,140). In the basolateral amygdala, it has been further demonstrated that the duration of the transient increase in spontaneous glutamate transmission depends on both severity and duration of stress, and is followed by a prolonged suppression in excitability (141).

### Ultradian rhythmicity is necessary for normal emotional processing in man

The molecular data above together with the behavioral experiments in rodents replaced with different corticosterone patterns provide compelling evidence that the amygdala is highly sensitive to dysregulated patterns of glucocorticoid exposure, but how does this translate into effects in the human brain. Using their block and replace protocol on healthy volunteers (135), Kalafatakis and colleagues also interrogated how different patterns of cortisol affected processing of emotionally salient information (134). Using a functional magnetic resonance imaging protocol assessing neural processing of emotional faces (IFEPT), the authors clearly demonstrated that different patterns of cortisol replacement had differential effects on the functional connectivity of areas underlying emotional processing including the amygdala, dorsal striatum, insula, and orbitofrontal cortex. This was reinforced by findings that nonpulsatile cortisol replacement was associated with a negative bias to ambiguous cues, and differences in recognition accuracy of emotional cues. These findings strongly support the notion that glucocorticoid rhythmicity modulates the neural dynamics underlying mood and anxiety.

## Altered HPA Dynamics in Disease

### Cushing’s syndrome

Cushing’s syndrome (CS) is characterized by excess levels of circulating glucocorticoid, most commonly due to exogenous treatment, which will be discussed in detail further into this review. More rarely, but posing a clinically important problem, CS is caused by unregulated excessive production of ACTH by a pituitary tumor (Cushing’s disease) (142,143), by ectopic secretion of ACTH, by cortisol secreting adrenal cortical tumors, or by multiple hypersecreting nodules in both adrenal cortices (primary macronodular adrenocortical hyperplasia) (144,145). While benign apparently nonsecreting adrenocortical tumors (incidentalomas) are found in up to 7% of the population (146), it is becoming clear that a proportion of these do have dysregulated cortisol secretion which can result in mild—but probably significant—hypercortisolemia (146).

Along with myriad metabolic adverse effects and cardiovascular risks (147), patients with CS demonstrate deficits in memory, and a wide range of cognitive impairments and mood disorders (148,149) as well as predisposition to anxiety and depression (150). This is most marked in older patients and in females (151,152). Although there is some preservation of pulsatility in Cushing’s patients, the 24-hour secretory pattern is characterized by an absence of the normal circadian variation and a failure of the plasma cortisol level to fall below 2 µg/dL between 23.00 and 03.00 hours (153).

### Obstructive sleep apnea

Obstructive sleep apnea (OSA) is a condition associated with increased prevalence of cardiovascular disease, metabolic syndrome and glucose intolerance (154), all symptoms associated with glucocorticoid excess. Henley et al (155) used frequent automated blood sampling in OSA patients, and found a marked disruption in HPA activity with significantly longer duration of ACTH and cortisol secretory episodes and larger pulsatile hormone release. Patients were reassessed after 3 months of compliant continuous positive airway pressure therapy, and HPA dynamics were found to normalize compared with untreated OSA. This study highlighted some important points about regulation and dysregulation of this highly dynamic system, as well as its recovery. It was hypothesized that one of the likely mechanisms underlying OSA-related HPA dysregulation was the potential damage that sleep disruption and hypoxic episodes can cause to the hippocampal formation, a major integrator of HPA negative feedback. Hippocampal neurons are highly susceptible to hypoxic/metabolic insults, and OSA patients exhibit hippocampal damage and cognitive deficits as a result (156). OSA patients also exhibit sympathetic nervous system hyperactivity (157), which can directly act on the adrenal cortex to alter adrenal responsiveness (158), as well as alter hippocampal corticosteroid receptor expression levels (159). The resulting net effect may be impaired rapid negative feedback, longer secretory episodes and as a result higher nadir levels of cortisol between individual pulses of cortisol release (160).

### Depression

According to the corticosteroid receptor hypothesis of depression (160) changes in the set point of the HPA axis are found in many patients with depression. In particular, altered regulation of ACTH and cortisol secretory activity, along with impaired corticosteroid receptor signaling have been postulated to underpin depressive psychopathology (161,162). In patients where neuroendocrine abnormality persisted, risk of relapse or resistance to treatment was much higher (163,164), supporting the validity of “psychoneuroendocrine” strategies (167–169). However, using a 24-hour frequent automated blood sampling protocol, Elizabeth Young (12,168) found that only 24% of a patient cohort of 25 depressed premenopausal women exhibited hypercortisolemia. In comparisons with matched controls, there was no difference in mean cortisol either between the patient group as a whole or those patients meeting criteria for atypical depression. Only the patients meeting criteria for endogenous depression showed increased cortisol. Reports of enlarged adrenal glands (169,170), and impaired negative feedback in depressed patients (171,172) have led to the theory that the level of impairment is at the GR-dependent negative feedback, either centrally or the level of pituitary. Treatment interventions have included CRH receptor antagonists (173), GR antagonists (174,175), and cortisol synthesis inhibitors (176,177). More recently, targeted antagonism of the GR chaperone protein FKBP51 (178,179) with a selective inhibitor (180,181) has proved promising.

### Infection, trauma, critical care medicine, and the inflammatory response

In healthy individuals, adrenal glucocorticoids exert anti-inflammatory and immune modulating actions (182–184). The acute increase in adrenal glucocorticoid secretion in response to infection, injury or trauma is therefore thought to be an adaptive homeostatic mechanism to prevent immunological over-reaction (185). Proinflammatory cytokines are rapidly induced in response to infection, injury or trauma and act to induce systemic or localized immune response. These factors also act directly on the adrenal gland in a mechanism signaling the body’s requirement for increased production and secretion of glucocorticoid hormone (186,187). Activated GR then acts in a well-characterized mechanism termed transrepression to directly switch off proinflammatory genes and inhibit the inflammatory actions of the cytokines, at target sites.

**Figure 8.**
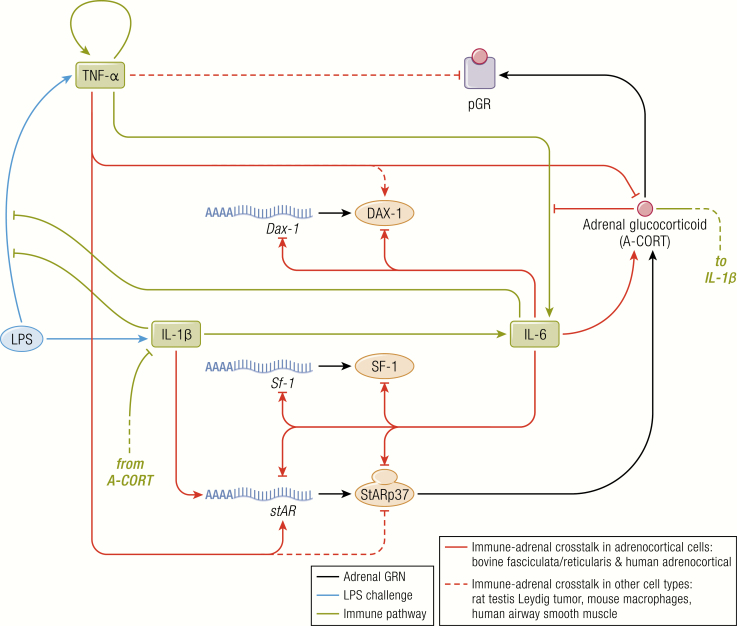
Crosstalk between the adrenal steroidogenic regulatory network and the immune pathway. During the inflammatory response induced by LPS, the synthesis of glucocorticoids in adrenocortical cells is modulated by the immune pathway through cytokines. The SRN, in turn, feeds back upon the cytokines signaling pathways. The Spiga et al. mathematical model has therefore also integrated these cytokine effects on the steroidogenic response to LPS. Their model predicts sustained induction of adrenal glucocorticoid (A-CORT) and inhibition of pGR, transient induction of *SF-1* and *StAR* transcription, transient inhibition of DAX-1 gene expression, and SF-1, StAR, and DAX-1 mRNA and protein dynamics that were then shown experimentally in LPS treated rats. From (69). Spiga F, Zavala E, Walker JJ, Zhao Z, Terry JR, Lightman SL. Dynamic responses of the adrenal steroidogenic regulatory network. Proc Natl Acad Sci U S A 2017;114:E6466–E6474. CC-BY OA. 2017, Springer Nature

An excellent example of the role of plasticity within the HPA axis is the response to coronary artery bypass graft surgery. Gibbison et al. (188) used perioperative automated blood sampling to measure cortisol at 10-minute intervals and ACTH at 60-minute intervals over a 24-hour period. ACTH and cortisol both initially rose to extremely high levels during the final stages of the surgery, but ACTH returned toward preoperative levels after the surgery was completed while supraphysiological cortisol pulses continued until the end of the 24-hour sampling time. While coordinated timing of ACTH and cortisol pulses was maintained in all individuals, the sensitivity of the adrenal gland to ACTH appeared to increase markedly after surgery so that very high levels of cortisol were produced despite low basal ACTH levels, a phenomenon which had been previously noted (189) but not interrogated with the detailed methodology of frequent blood sampling (190) or at the mechanistic level until the study of Gibbison and colleagues (188). The mechanism suggested for this striking adrenal hypersensitivity is mediated by systemic inflammatory signals (193–195) inducing cytokine-mediated sensitization of adrenal responses to ACTH (194). In support of this, rats treated with the potent activator of immune response, LPS, exhibit similarly increased adrenal sensitivity resulting from changes in the regulation of both stimulatory and inhibitory intra-adrenal signaling pathways (69) as shown in Fig. 8. Whether the ACTH cortisol dissociation after coronary artery bypass graft surgery is a solely cytokine mediated effect or acts in concert with a combination of factors is the subject for further study (195) but it provides an excellent example of the body’s survival mechanisms at play.

This highly advantageous adaptive process of immune modulation of adrenal glucocorticoid secretion (187) also unfortunately has the potential to become maladaptive in cases of chronic inflammatory disease (196,197). In an experimental rat model of adjuvant-induced arthritis, corticosterone dynamics are severely affected (198). Higher frequency pulses are detected throughout the 24-hour period, with a loss of the normal circadian nadir in corticosterone secretion (47). This form of hyperactive HPA axis has similarities to the high amplitude pulses detected in rats subjected to 6 weeks of 200 lux bright light (61), which is a chronic stressor for the nocturnal animals as well as disrupting their circadian cues. There are no parallel studies looking at 24-hour levels of cortisol in man, and although studies of cortisol levels in rheumatoid arthritis patients have not always produced consistent results, there is evidence that plasma cortisol levels may be elevated with a loss of circadian nadir and reduced reactivity to HPA axis stimulation (199,200). This raises the important question of whether chronic inflammation induces a form of glucocorticoid resistance, which then creates a vicious cycle in disease progression.

## Treatment with Synthetic Glucocorticoids

Synthetic glucocorticoids are one of the most commonly prescribed classes of drugs, primarily for treating inflammatory conditions, from skin conditions such as dermatitis to rheumatological diseases such as systemic lupus erythematosus, Crohn’s disease, ulcerative colitis, rheumatoid arthritis and asthma. In the UK and US, nearly 2% of the adult population use oral glucocorticoids at any given time (201,202). Treatment with synthetic glucocorticoids results in raised levels of glucocorticoids throughout the day with a loss of both ultradian and circadian oscillations (134). Side effects are multiple, across metabolic, skeletal, cardiovascular, and immune systems, but also affecting the central nervous system with patients often complaining of changes to mood, with a proportion of these reporting bouts of depression, and in severe cases, mania (203). Interestingly, reports of depression and psychiatric illness reported with synthetic glucocorticoid use, are not dissimilar to those reported by patients with Cushing’s disease and CS, who present with elevated levels of glucocorticoid hormones throughout the day (204,205). Sleep disturbances are commonly reported and studies have revealed that memory is significantly impaired with long-term prednisolone treatment (206–209).

## Potential for Chronotherapy: A Pulsatile Replacement Strategy in Addison’s Disease as Proof of Principle

Prior to the development of steroid replacement therapy, adrenal insufficiency resulted in 10% mortality rates (210). Glucocorticoid replacement had a miraculous effect—but it is often overlooked that standard replacement therapy is also associated with an increased mortality, with a standardized mortality ratio over 2. Furthermore, standard replacement therapy results in impaired health-related quality of life (211–216), adverse metabolic and cardiovascular risk profiles (217), increased levels of proinflammatory cytokines (218), and—most disabling for many patients—reduced activity, low motivation, and mental fatigue with associated high levels of unemployment and disability benefits (214,219–223).

We are now at a critical point for the development of safer and more effective glucocorticoid replacement therapy. One way forward is the development of physiological pulsatile replacement therapy using miniature nanopumps or subcutaneous reservoirs. We have shown proof of principle that subcutaneous infusions can reproduce normal circadian and ultradian rhythmicity (224). Furthermore, we have gone on to compare responses of normal volunteers to physiological circadian rhythms of cortisol either with or without pulsatility of the infused cortisol (134). In this study the pulsatile infusions promoted better quality of sleep, improved performance of working memory, and also resulted in differential effects on attentional bias to and recognition of emotional cues. This was also associated with differential responses in functional connectivity of brain regions which process emotional responses. The importance of these effects in longer term replacement therapy in patients with Addison’s disease is currently under investigation.

It is not only replacement therapy that might benefit from ultradian chronotherapy. Glucocorticoid hormones are currently prescribed to over 1% of the population (201) and side effects are apparent even after short-term (< 30 days) duration (225), while even “side-effects sparing corticosteroids” cause weight gain, the metabolic syndrome and adverse cardiometabolic effects (226). It is becoming clear that we need to improve steroid therapy and whether the administration of oscillating levels of glucocorticoids can modify the ratio of wanted effects to unwanted side-effects needs to be investigated.

## Abbreviations

ACTH: adrenocorticotropin
AF1: activation function 1
AF2: activation function 2
CA: cornu ammonis
CRH: corticotropin-releasing hormone
CS: Cushing’s syndrome
DBD: DNA binding domain
DNA: deoxyribonucleic acid
GFP-GR: green fluorescent protein tagged GR
GR: glucocorticoid receptor
GRE: glucocorticoid response element
HC: hippocampus
HPA: hypothalamic–pituitary–adrenal
LBD: ligand-binding domain
mPFC: medial prefrontal cortex
MC2R: melanocortin type-2 receptor
MR: mineralocorticoid receptor
NP: nonpregnant
NTD: N-terminal domain
PVN: paraventricular nucleus
SCN: suprachiasmatic nucleus
ZF: zona fasciculata
